# Old foes following news ways?—Pandemic-related changes in the epidemiology of viral respiratory tract infections

**DOI:** 10.1007/s15010-023-02085-w

**Published:** 2023-08-29

**Authors:** Nicole Maison, Jimmy Omony, Sophia Rinderknecht, Laura Kolberg, Melanie Meyer-Bühn, Erika von Mutius, Johannes Hübner, Ulrich von Both

**Affiliations:** 1 Department for Asthma and Allergy, Dr Von Hauner Children’s Hospital, LMU University Hospital, LMU Munich, Lindwurmstr. 4, 80337 Munich, Germany; 2 Institute for Asthma- and Allergy Prevention (IAP), Helmholtz Zentrum Munich, German Research Center for Environmental Health (GmbH), Munich, Germany; 3 Department of Infectious Diseases, Dr Von Hauner Children’s Hospital, LMU University Hospital, LMU, Munich, Germany; 4 German Center for Lung Research (DZL), Munich, Germany; 5 German Center for Infection Research (DZIF), Partner Site Munich, Munich, Germany

**Keywords:** Respiratory tract infection, Pandemic, Epidemiology, Rhinovirus, Respiratory viruses

## Abstract

**Introduction:**

Following lockdown periods and restricting public health measures in response to the COVID-19 pandemic, respiratory tract infections (RTIs) rose significantly worldwide. This led to an increased burden on children’s hospitals compromising medical care of acutely and chronically ill children. We characterized changes in the epidemiological pattern of circulating respiratory viral infections.

**Methods:**

We assessed the number of patients with RTIs and the annual distribution of virus detections between 2019 and 2022 based on 4809 clinical samples (4131 patients) from a German pediatric tertiary care-center. We investigated the impact of lockdown periods on spectra of circulating respiratory viruses, pattern of coinfections, age, and seasonality of infections.

**Results:**

A fourfold increase in the number of respiratory virus detections was observed in 2022 vs 2019 with numbers doubling in 2022 (vs 2021). In 2022, seasonal patterns of circulating virus, particularly Adeno and seasonal Coronavirus were far less pronounced compared to previous years, in fact almost disappeared for Rhinoviruses.”. SARS-CoV-2, Parainfluenza- and human Metapneumovirus detections increased significantly in 2022 (2019 vs 2022, p < 0.01). Coinfections with multiple viruses occurred more frequently since 2021 compared to pre-pandemic years, especially in younger children (2019 vs 2022, p < 0.01).

**Conclusion:**

Compared to pre-pandemic years, we observed a dramatic increase in pediatric RTIs with an incrementing spectrum of viruses and a predominance in Rhino/Enterovirus infections – leading to a high rate of hospital admissions, particularly in conjunction with other viruses. This caused an acute shortage in medical care and may also be followed by an increase of virus-triggered secondary chronic respiratory diseases like asthma—rendering a burden on the health system.

**Supplementary Information:**

The online version contains supplementary material available at 10.1007/s15010-023-02085-w.

## Introduction

Respiratory tract infections (RTIs) are among the most common childhood illnesses resulting in a high number of physician contacts and hospitalizations. RTIs contribute decisively to morbidity and mortality in the early years of life [1]. Infections with respiratory syncytial virus (RSV) and Rhinovirus type A and C are associated with chronic lung disease, like asthma, later in life [2–4]. RTIs are characterized by an epidemic occurrence at virus-specific seasonal times [5, 6]. Climatic factors such as temperature and humidity affect respiratory virus stability and transmission rates, and appear to affect the host’s intrinsic, innate, and adaptive immune responses to RTIs. This may explain why most viral infections were observed in the fall and winter seasons [7, 8]. However, human behavioral patterns are a crucial factor in the spread of viruses influencing contact rates between infected individuals and susceptible hosts. With the onset of the COVID-19 pandemic, contact rates were significantly reduced to contain the spread of SARS-CoV-2. In addition, NPI such as the use of masks and hand hygiene significantly reduced the transmission of viruses. This led not only to containment of the pandemic but also to a significant decrease in other viral infections, such as Influenza A/B and RSV [9, 10]. After easing of pandemic-related measures, a rising and persistent wave of non-COVID-19 RTIs in children was observed in various countries since June 2021 [11–14].

After initially assuming that respiratory viruses would only occur outside of their typical season, it quickly became apparent that the number of infected children was also increasing [12]. The actual impact on epidemiological changes that occurred by the end of 2022, the consequences and potential causes of these changes are not well-understood [15].

At the beginning of the pandemic, infections with SARS-CoV-2 played only a minor role in children. From autumn 2021, with the beginning of the circulation of the omicron variant, younger patients got increasingly infected [16]. Initially, only mild acute infections occurred, with admissions to children’s hospitals increasing from September 2021. Agathis et al. described that patients with co-detection of other respiratory viruses other than RSV and Rhino/Enterovirus (RV), showed a more severe course of disease [17].

Although several European countries reported an increase in RTIs in 2022, there is hardly any data as to whether the frequency and clinical impact of coinfections differed compared to the pre-pandemic period. It remains unclear whether this development will only remain a short-term phenomenon. It is equally unclear whether children with severe courses of acute infection will have to fear long-term consequences. We know that infections with RV or RSV, even if they take a mild course, significantly increase the risk of respiratory complications and chronic respiratory illness [4].

In this study, we aimed to illustrate the spectra of circulating viruses, patterns of coinfections and age and seasonality of RTIs in children from 2019 to 2022. We set out to provide a better understanding of pandemic-related changes in the epidemiology and the interaction of viral respiratory diseases—whose impact has since increased drastically on the population and healthcare system.

## Methods

At the Hauner Children’s Hospital (Munich), a large tertiary care university hospital, the number of detected viral infections in patients between January 2019 and November 2022 was investigated. We analyzed 4702 (2019 with n = 550, 2020 with n = 735, 2021 with n = 1243, and 2022 with n = 2243) positive viral results from routine clinical specimens of both outpatients and inpatients (n = 4131). We included all patients presenting as outpatients or inpatients in the defined study period that had a positive test result at the in-house microbiology laboratory for at least one of the following viruses: Influenza A/B, RSV, Parainfluenza virus, human Metapneumovirus, Adenovirus, seasonal Coronaviruses (OC43, NL63, E229, HKU1) SARS-CoV-2, and Rhino/Enterovirus. Testing was performed at the discretion of the attending physician when an acute respiratory illness was diagnosed requiring either inpatient admission or outpatient drug treatment. Particular criteria were: reduction in oxygen saturation below 92%, wheezing, fever, poor general condition or young age of the patient. During the COVID-19 pandemic, multiplex-PCR testing (including RSV, Influenza A/B, and SARS-CoV-2) was performed on patients regardless of specific respiratory symptoms in case of hospital admission. Samples were taken by nasal or pharyngeal. All samples were tested by immunofluorescence assay (Quidel Sofia 1) [18] or using of a Mulitplex PCR system; (BioMerieux Biofire, Askim, SWEDEN [19]; Extraction by Nimbus and subsequent analysis by BioRad CFX, Seegene PCR-Kit) [20].

### Statistical analysis

The Wilcoxon’s rank test was used to compare categorical (grouped) variables. The Pearson’s Chi-square test (using Cramer’s V) was used to evaluate for the goodness of fit between variables. The Z-test was used to compare proportions of the number of positive tests for individual virus groups across gender (males vs females). The analysis we assumed independence between the categorical (or grouped) variables. The variables included in the Chi-square test were gender (males vs females), age-groups (< 6, 6–12, and ≥ 12 years of age), virus infection mode (as binary: single vs multiple infections). Single virus infections were more prominent among children from the younger age-groups (< 6, 6–12, ≥ 12 years), so we performed a Chi-square test for independence to assess if the infection mode is independent of the age-group. Proportionally fewer subjects with coinfections (“multiple”) were observed in the pre-pandemic year (2019) compared to the pandemic years (vs 2020, vs 2021, vs 2022). The Kruskal–Wallis test was used to test for differences in the overall mean age of children across four years. In the post-hoc analysis, differences in the mean age of children between the four years (pairwise comparisons) were done using the Wilcoxon’s rank test. The data was curated in Microsoft Excel and analyzed in R software [21].

### Ethics approval

The Ethics committee of the Ludwig-Maximilians University (LMU) Munich approved this study under project numbers 21-0334 and 23-0061.

## Results

### Prevalence of RTIs from 2019–2022

**Fig. 1:**
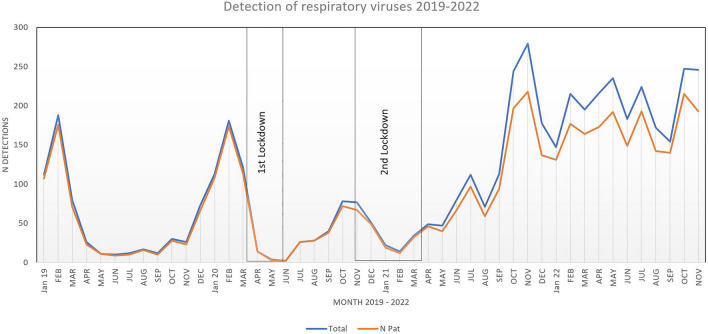
Monthly distribution of respiratory viruses detected, including Influenza A/B, RSV, Parainfluenzavirus, Metapneumovirus, Adenovirus, seasonal Coronaviruses, SARS-CoV-2 and Rhinovirus (blue) and number of patients infected (orange), years 2019–2022

**Fig. 2:**
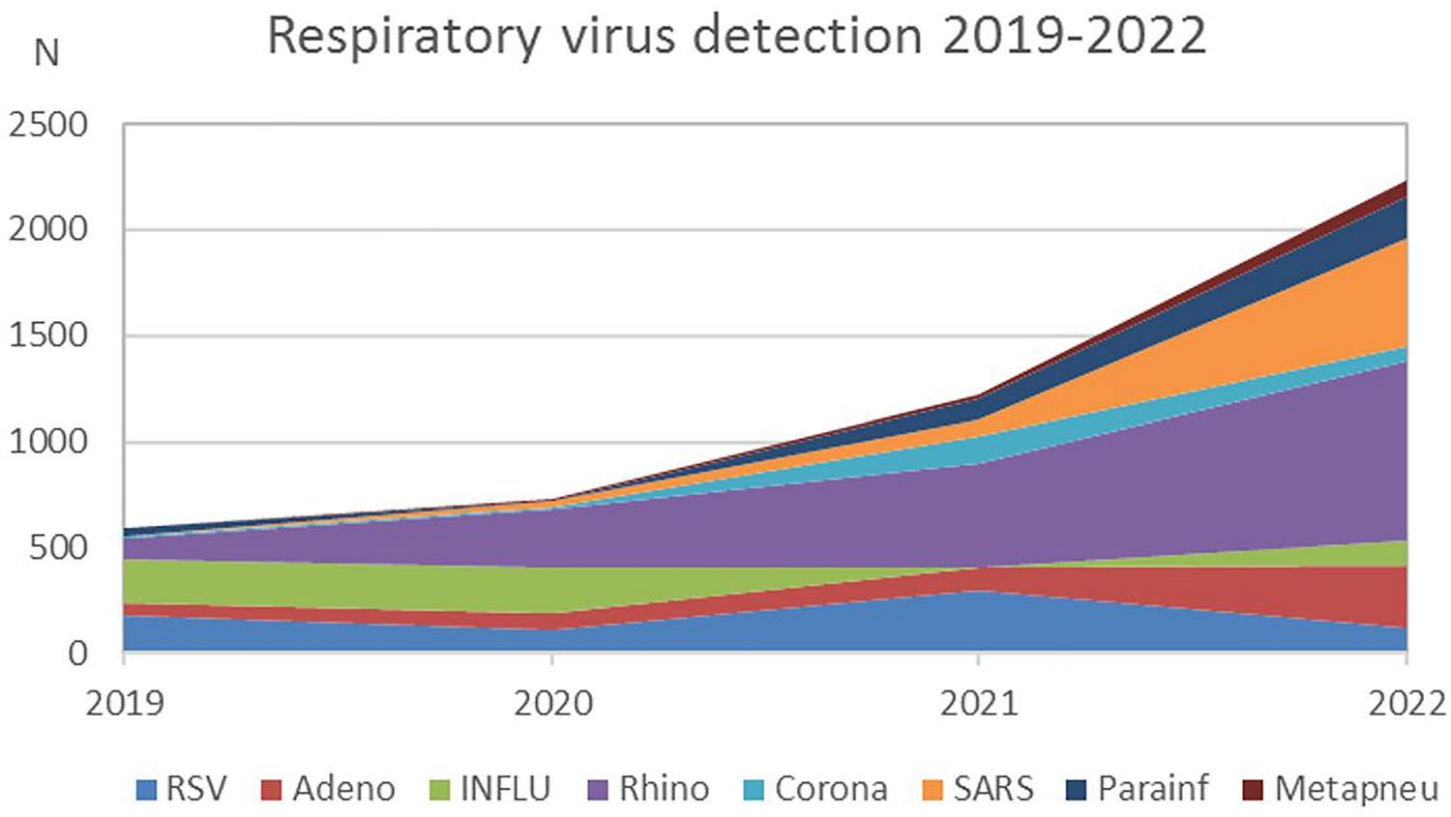
Cumulative numbers of respiratory virus detections (years 2019–2022)

In pre-pandemic years (2017–2019), we demonstrated stable numbers of respiratory virus detections following a seasonal pattern [11]. As previously described, changes in the epidemiology of respiratory infections occurred in the context of introducing pandemic hygiene measures. These led to a significant reduction in circulating respiratory viruses, followed by a marked increase in circulating respiratory viruses after the first and second lockdowns [11]. Comparing the total numbers for respiratory virus with 2019, an increase of 23% in 2020, 100% in 2021 and 270% in 2022 was observed (Table 1). An all-time high in respiratory virus detection was observed in November 2021, with a decline to pre-pandemic year levels in December 2021 and January 2022. While both the pre-pandemic and the lockdown periods (1 & 2) showed relatively low numbers in March/April 2019, 2020 and 2021, consistently high detection rates occurred from May 2021 onwards, with significantly higher numbers across all detected viruses (Fig. 1). RV accounted for the highest proportion of infections, followed by SARS-CoV-2, Adenovirus, RSV and Influenza virus. In addition, Parainfluenza- and Metapneumovirus showed increased case numbers (Fig. 2).

**Table 1:** Numbers of virus detections and patient characteristics from January 2019 to November 2022. Mean age comparison pairwise comparisons between viruses using Wilcoxon’s test

	2019	2020	p-value 2019 vs 2020	2021	p-value 2019 vs 2021	p-value 2020 vs 2021	2022	p-value 2019 vs 2022	p-value 2020 vs 2022	p-value 2021 vs 2022
RSV, n	184	113		301			124			
Age (mean)	1.43	1.45	0.99	2.17	0.016	0.037	2.95	0.0015	0.0039	0.16
Sex (% male)	61.4	58.4	0.695	55.1	0.208	0.629	56.1	0.418	0.821	0.944
Adeno	55	75		106			298			
Age (mean)	4.8	3.49	0.12	3.75	0.25	0.49	3.7	0.26	0.44	0.89
Sex (% male)	61.5	57.8	0.829	58.9	0.882	1.00	53	0.324	0.576	0.352
Influenza A/B	204	223		3			114			
Age (mean)	4.36	4.7	0.29	7.28	0.1	0.19	7.12	< 0.001	< 0.001	0.96
Sex (% male)	51.5	61.9	0.037	40	0.957	0.596	45.6	0.377	0.006	1.00
Rhinovirus	101	273		488			846			
Age (mean)	4.15	3.88	0.9	4.02	0.84	0.99	4.1	0.89	0.87	0.84
Sex (% male)	75.2	62.6	0.031	58.3	0.002	0.277	54.9	< 0.001	0.032	0.225
Corona	15	9		128			70			
Age (mean)	1.37	5.79	0.0019	4.18	0.002	0.13	4.48	0.0024	0.23	0.62
Sex (% male)	73.3	75	1.00	62.9	0.610	0.756	54.3	0.286	0.457	0.306
SARS-COV-2	0	25					508			
Age (mean)	NA	5.93	NA	6.35	NA	0.87	4.46	NA	0.12	0.055
Sex (% male)	NA	68	NA	58	0.166	0.50	48.4	0.506	0.088	0.125
Parainfluenza	35	4					194			
Age (mean)	3.82	8.38	0.023	2.57	0.26	0.0046	3.98	0.8	0.022	0.016
Sex (% male)	88.9	NA	NA	50.7	0.070	1.00	52.8	0.075	0.474	0.873
Metapneumo	3	13					80			
Age (mean)	1.03	5.42	0.043	3.21	0.31	0.069	2.62	0.3	0.012	0.99
Sex (% male)	100	1	NA	58.3	0.733	1.00	62.5	0.731	1.00	1.00
Total n	597	735					2234			
Age (mean)	3.4	3.9	0.024	3.8	1	0.092	4.4	< 0.001	1	0.005
Sex (% male)	57.9	61.7	0.901	57.5	0.158	< 0.001	52.8	0.083	< 0.001	0.01
No. patients (N)	550	694					1869			

Mean age of all patients comparing 2019–2022 using chi-square (3 df) = 25.32, p < 0.0001, with n = 4131

To determine the significance of virus detections in relation to the number of hospitalized patients, admission data from 2019 to 2022 were analyzed. Patients whose primary treatment diagnosis was coded with upper respiratory tract infections (URTI) (ICD-10 J00-J06), influenza/pneumonia (ICD-10 J09-J18), and lower respiratory tract infection (LRTI)/ acute bronchitis (ICD-10 J19-J20) were included. We observed a 22% reduction in hospital admissions in 2020 vs 2019 and an increase of 11% in hospital admission comparing the post-lockdown (2022) with the pre-lockdown period (2019). In addition, an increase of upper respiratory tract infections post-lockdown vs pre-lockdown (2021 vs 2019: 17%; 2022 vs 2019: 30%) and an increase of lower respiratory tract infection post-lockdown vs pre-lockdown (2021 vs 2019: 25%; 2022 vs 2019: 7%) was seen. There was no change in influenza/pneumonia pre- vs post-lockdown; less influenza/pneumonia during lockdown was observed (Supplementary Table E1).

### Changes in the seasonal pattern of respiratory viruses

**Fig. 3:**
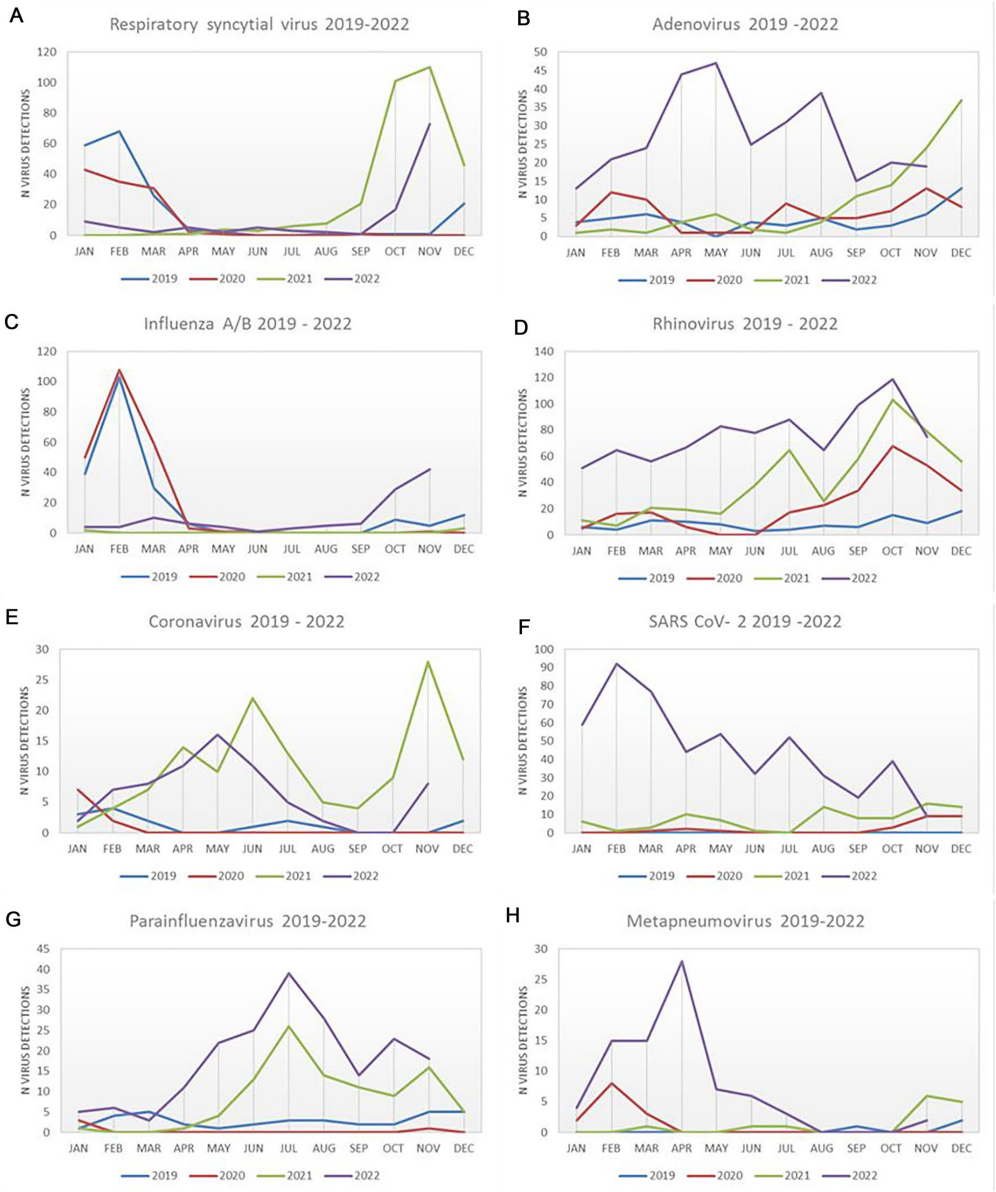
Seasonal pattern of number of respiratory virus infections **A** Respiratory syncytial virus, **B** Adenovirus, **C** Influenza **A**/**B**, **D** Rhinovirus, **E** Seasonal Coronavirus, **F** SARS-CoV-2, **G** Parainfluenza, **H** Metapneumovirus

In pre-pandemic years the season for RTIs in the Munich area would start in September and end in March; however, consistently high numbers for RV, Adenovirus and Metapneumovirus were observed in summer 2022 (Figs. 1 and 3B,D,F). We observed distinct changes in the seasonal infection pattern for each virus in 2021 and 2022.

Infections with Parainfluenza virus were barely detected outside the winter period in pre-pandemic years. However, 2021 and 2022 demonstrated rising numbers of detected viruses with peaks in July 2021/2022. A high number of human Metapneuomovirus infections occurred in April 2022, while in previous years, this virus did not play any particular role in children. Seasonal coronaviruses showed peaks in winter month and around July in pre-pandemic years. In contrast, in 2022, high numbers were observed in May, June and November. SARS-CoV-2 caused only a few relevant childhood infections in the first two pandemic years 2020/2021. In 2022, however, numerous infections occurred, peaking in January, and slowly declining over the summer months. Adenovirus infections became increasingly relevant from September 2021 onwards. The total number of infections remained significantly higher in 2022 compared to the previous years, with a maximum in July and August 2022. In 2019 and 2020, typical Influenza A/B waves were observed from January to March – which were absent in 2021. However, in autumn 2022, increasing numbers of cases occurred well before the typical season (pre-pandemic) for Influenza A/B infection in the region.

The typical infectious season for RSV spreads from November to February, as observed in pre-pandemic years. This changed significantly during the pandemic with infections occurring much earlier and with significantly higher case numbers – with cases detected as early as August 2020. A similar pattern was observed in 2022 with increasing numbers from September. Particularly impressive changes were seen for RV detections. In contrast to all other respiratory viruses, which varied seasonally, Rhino/Enterovirus infections were consistently high across all months in 2022 (Fig. 3).

### Coinfections and association between age /gender and type of virus infection

**Fig. 4:**
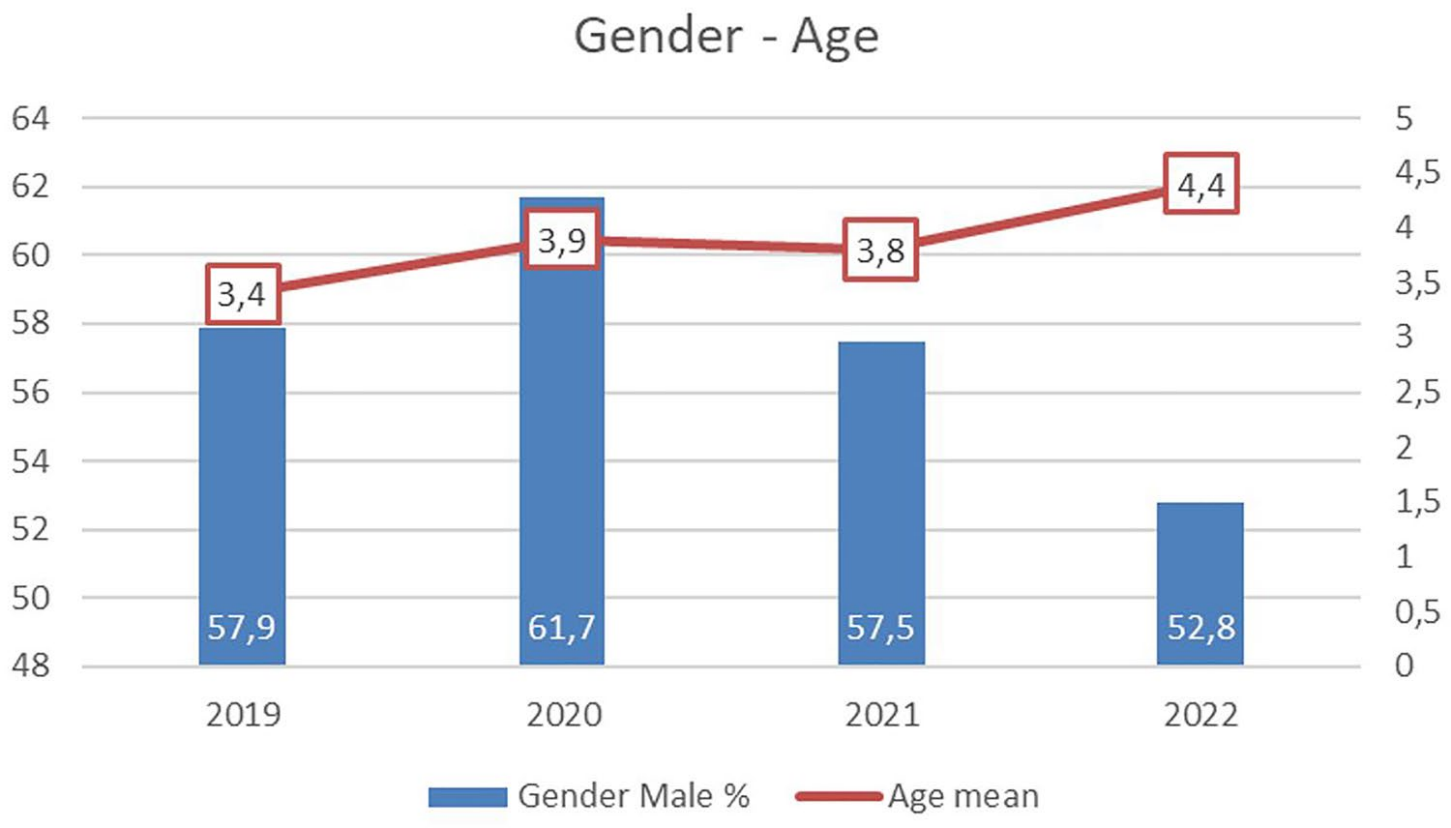
Percentage of male and mean age in years of patients with viral respiratory tract infections (years 2019–2022)

Compared with pre-pandemic years, there were significant changes in age (2019 vs 2022 p < 0.001) and sex-related association with respiratory virus infection (2020 vs 2022 and 2021 vs 2022 p < 0.001) in children, with an increase in female and older children (Fig. 4).

Children infected with RSV, seasonal Coronavirus, Influenza A/B, and Metapneumovirus predominantly drove this observation. On the other hand, the number of infections with RV was not associated to the age of affected children. The proportion of females increased for RV infections, and this was also the case for Influenza infections (Table 1). A significant increase in coinfections was observed in 2021 and 2022 (2019 vs 2021 and 2019 vs 2022 p < 0.001) compared to 2019 (Table 2).

**Table 2:** Numbers and proportion of respiratory viral co-infections per year (years 2019 – 2022)

	2019		2020		2021		2022		Total		P-Value		
	n	%	n	%	n	%	n	%	n	%	2019 vs 2020	2019 vs 2021	2019 vs2022
Single	501	0.91	655	0.94	818	0.8	1559	0.83	3533	0.86	0.003	0.00	0.00
Multiple	49	0.09	39	0.06	200	0.2	310	0.17	598	0.14			
Total	550	1	694	1	1018	1	1869	1	4131	1			

**Fig. 5:**
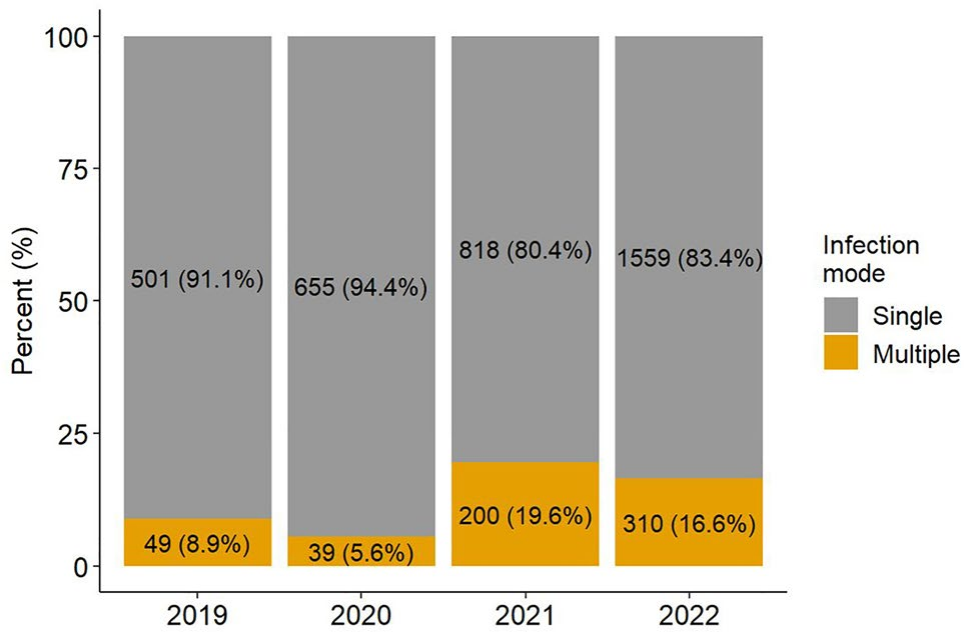
Percentage of single and multiple virus detections, years 2019–2022

In 2019, detection of two viruses in one sample was observed in only 8.9%, this share doubled in 2021 (19.6%) and was 16.6% in 2022 (Fig. 5).

**Fig. 6:**
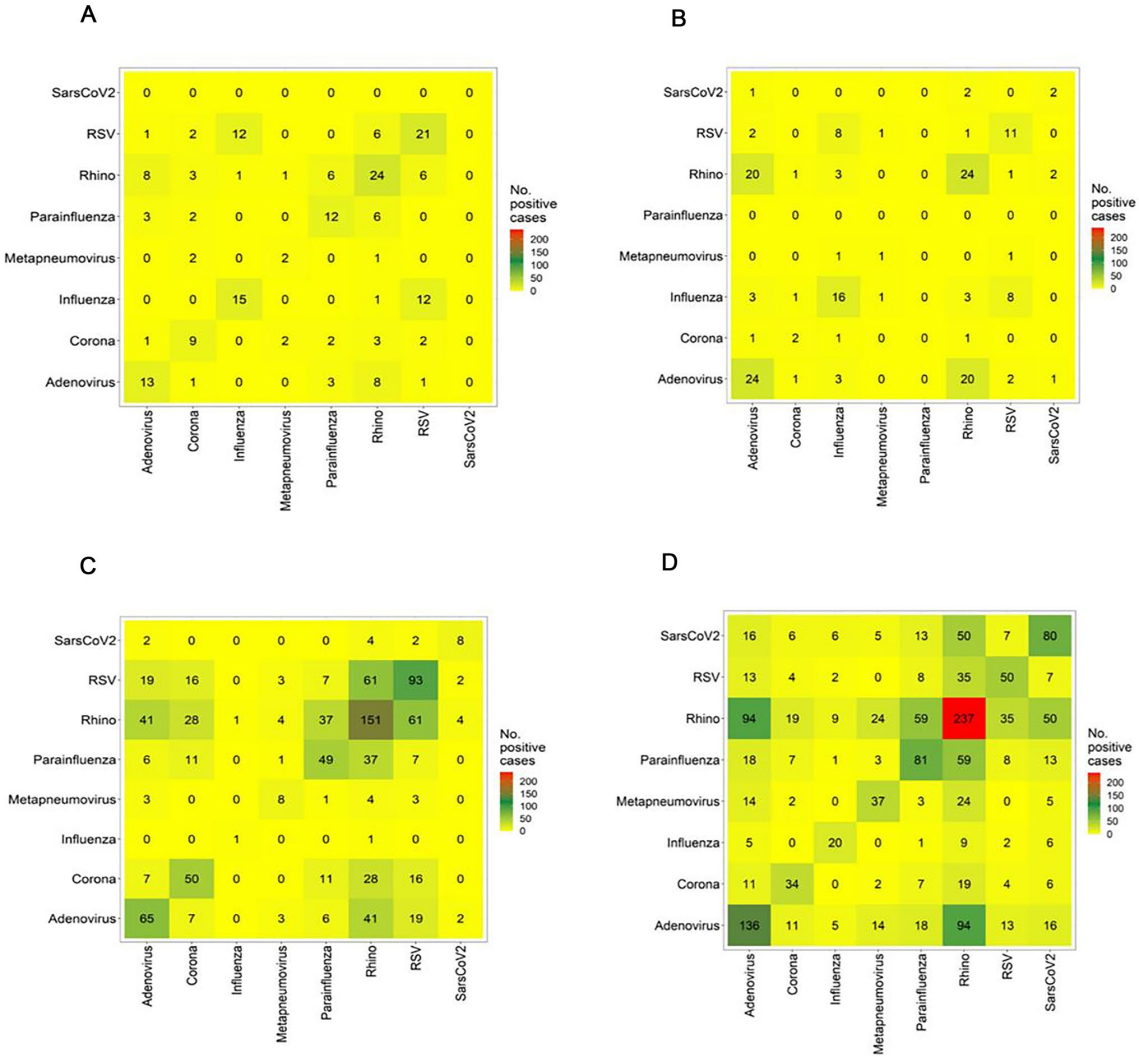
Heat map diagram showing respiratory viral co-infections: co-infections occurred most frequently in 2022, in combination with Rhinovirus and Parainfluenza, SARS-CoV-2 or RSV, and SARS-CoV-2 with RSV

More than two viruses were observed in less than 1% in 2019 and 2020, and occurred in 5–6% of cases in 2021 and 2022. In 2021, up to four viruses per sample were detected for the first time, and in 2022 in individual cases, up to five viruses were detected in a single sample (Table E). Coinfections of RV and SARS-CoV-2 occurred significantly more frequently in younger children (age 0–2 years), and in 2022 in RV and Adenovirus infections (Figure E1, E2). Most coinfections occurred in the context of a RV infection. In 2021, RV coinfections were particularly detected frequently with Parainfluenza or RSV. Coinfections with Metapneumovirus and SARS-CoV-2 were also observed in 2022 (Fig. 6).

## Discussion

In this study, we observed a dramatic increase in pediatric RTIs with an incrementing spectrum of viruses and an astonishing rise in RV infections leading to a high rate of hospital admissions in 2022. Hospitalizations due to upper and lower respiratory tract infections were observed more frequently in the post-lockdown than in the pre-lockdown period. There were significant changes in the age of affected children and a notable variation in the association between virus infections with sex in 2021–2022 compared to the pre-pandemic year 2019. More female and older children were particularly affected. The annual numbers of coinfections changed markedly, especially in younger children infected with RV.

Epidemics involving viral RTIs have always vary in regional intensity and duration—This is thought to be due to the genetic diversity of circulating viruses and their respective virulence. Climatic changes also seem to impact on virus spread [7, 22, 23]. Numerous studies have shown that NPIs reduce the circulation of SARS-CoV-2 and other respiratory pathogens [24]. While certain non-respiratory infections, such as Rotavirus, occurred less frequently in times of hard lockdowns, a rebound of Rota- and other viruses were already evident when NPIs were still in place. Consistent with our data, Engels et al. reported that Adeno- and Rhino/Enteroviruses detections increased as early as the second and third COVID-19 waves [25]. Interestingly, these are non-enveloped viruses, rendering them less sensitive to disinfection agents. Most other respiratory viruses are transmitted by aerosols. However, Rota-, Adeno- and Rhino/Enterovirus are also following a contact-transmission route and may therefore, be less preventable by the use of facemasks. This corroborates the observation in our study, that RV was highly present despite NPIs. Our results are supported by Buchholz et al.: The authors describe high numbers of RSV infection at the beginning of the year 2020, a sharp decline and low numbers during the lockdown periods and rising numbers in the end of 2021 and 2022. The observation that RV/EV was hardly affected by the hygiene measures and continued to lead to respiratory tract infections from 2020–2021 was also demonstrated. Consistent with our data, an overall increase in infections in 2022 was observed in their multicenter study, on observation that is complemented by our data covering an additional period until November 2022 [26].

We noted that the virus-specific seasonality observed in pre-pandemic years changed in the post-lockdown period. This, however, was not observed for all viruses but was predominantly prevalent for infections with RV, Adenovirus and Metapneumovirus. Host-specific properties of these viruses may also contribute to this finding. While RV is consistently detected in children, RSV is predominantly found in young children during epidemic phases [27]. Outside of epidemics, RSV was mainly detected in adults at risk (usually with COPD—chronic obstructive pulmonary disease) or children with immune deficiency [28, 29]. While RV was able to spread unhindered among children, the occurrence of RSV was long prevented by mandatory masking for adults, which reduced transmission events. This supports our observation that RSV only occurred more frequently after the lifting of the mask regulation in September 2022. Infections with RV, Parainfluenza, Metapneumovirus, and Adenovirus occurred despite the NPIs and were also detected more frequently than before the pandemic. The presence or absence of certain pathogens could have played a role.

In previous studies, virus-virus interactions could be disentangled, which caused a promoting or inhibiting effect on the pathogenicity of viruses [30, 31]. Greer et al. demonstrated that RV detection was associated with a reduced probability of detecting human Adenoviruses, Coronaviruses, Bocavirus, Metapneumovirus, respiratory syncytial virus, Parainfluenza virus and Influenza A virus [32]. A negative interaction was also observed between RV and SARS-CoV-2 by Dee et al., leading to the hypothesis that RV infections could reduce the number COVID-19 cases [33]. The presence of RV could therefore, reduce susceptibility for other RTIs. However, it remains unclear whether the absence of certain viruses influenced susceptibility and pathogenicity of the host towards RV. Contrary to this assumption, the data from our study shows an increase in co-detections, particularly in conjunction with RV. The significance of the increasing virus co-detections cannot be clarified with certainty at present. In chronically ill patients, who make up a large proportion of the inpatients in this study, RV in particular can represent a colonization rather than a trigger for the underlying complaints. Nevertheless, the significant increase in hospital admissions in 2022 in our data indicates a serious clinical impact of multiple detections of respiratory viruses in children.

In addition to the exposure to viral pathobionts, bacteria probably play an equally important role. Initial studies suggest that the reduced exposure to pathogens could have led to a change in the lung microbiome [34]. The development and stability of the microbiome of the respiratory tract are decisive factors in susceptibility to viral infections, which also determine the risk of developing chronic lung diseases such as asthma [35].

Frequent occurrence coinfections, particularly seen in young patients, were complemented by a change in the mean age of the affected children. In 2022, RSV infections were significantly more common in older children. This might be attributed to a lack of exposure to the virus in early childhood and to a lack in transfer of protective antibodies from unexposed mothers. Thus, an increase in the number of cases could be due to an accumulation of various infections in larger group of children including a wider age range who have missed out exposure to pathogens.

Finally, our data indicates that a combination of many confounding factors has caused the increase in detection rates and coinfections and changes in the seasonal pattern of viral RTIs. A limitation of this monocentric study is that it only draws a regional picture and does not allow deductive statements about the impact on the clinical course of the virus detections. This issue needs to be addressed in a follow-up study. Since test strategies have changed during the pandemic, a bias in the detection of viruses cannot be ruled out. However, while short-term consequences for the morbidity of children are already obvious, the impact on resulting chronic lung disease is unclear and remains the focus of further studies.

## Conclusion

COVID-19 pandemic-related interventions caused a profound change in the epidemiology of viral RTIs in children, with an increasing spectrum of circulating viruses and an astonishing peak in RV infections leading to a high rate of hospital admissions in 2022. Compared to pre-pandemic years, infections occurred more frequently in female and older children. The annual number of coinfections changed markedly – especially in younger children, predominantly in conjunction with RV. The underlying factors associated with the increase in cases, the peak in coinfections, and changes in the seasonal pattern of viral RTIs are not completely understood and remain the focus of further studies.

### Supplementary Information

Below is the link to the electronic supplementary material.

Supplementary file1 (DOCX 144 KB)

## Data Availability

The data of this ongoing study are currently not available for public use due to data protection reasons.

## Abbreviations

NPI: Non-pharmaceutical intervention
LRTI: Lower respiratory tract infection
RTI: Respiratory tract infections
RV: Rhino/Enterovirus
RSV: Respiratory syncytial virus
SARS-CoV-2: Severe acute respiratory syndrome coronavirus 2
URTI: Upper respiratory tract infection
