# Supplementary material for: Old foes following news ways?—Pandemic-related changes in the epidemiology of viral respiratory tract infections
Source: Infection. 2023 Aug 29;52(1):209–18. doi: 10.1007/s15010-023-02085-w (PMC10811157; doi:10.1007/s15010-023-02085-w)
Supplement: Supplementary file 1 — Supplementary file1 (DOCX 144 KB) [file 15010_2023_2085_MOESM1_ESM.docx]

**Supplemental Tables and Figures**

**Table E1:**

| **Month** | **Jan** | **Feb** | **Mar** | **Apr** | **May** | **Jun** | **Jul** | **Aug** | **Sep** | **Oct** | **Nov** | **Dec** | **total** |
| --- | --- | --- | --- | --- | --- | --- | --- | --- | --- | --- | --- | --- | --- |
| Upper respiratory tract infections (ICD J00-J06) | | | |  |  |  |  |  |  |  |  |  |  |
| **2019** | 10 | 15 | 16 | 9 | 5 | 7 | 10 | 7 | 15 | 15 | 12 | 20 | 141 |
| **2020** | 16 | 16 | 11 | 2 | 5 | 4 | 12 | 6 | 15 | 15 | 10 | 14 | 126 |
| **2021** | 5 | 3 | 17 | 14 | 14 | 19 | 13 | 10 | 13 | 19 | 25 | 13 | 165 |
| **2022** | 14 | 10 | 8 | 15 | 23 | 22 | 29 | 8 | 9 | 21 | 12 | 13 | 184 |
| Influenza/Pneumonia (ICD J09-J18) | | |  |  |  |  |  |  |  |  |  |  |  |
| **2019** | 26 | 38 | 19 | 10 | 14 | 8 | 11 | 8 | 15 | 14 | 5 | 20 | 188 |
| **2020** | 31 | 49 | 22 | 7 | 6 | 3 | 10 | 11 | 8 | 5 | 9 | 4 | 165 |
| **2021** | 8 | 2 | 9 | 8 | 2 | 8 | 11 | 12 | 7 | 3 | 18 | 11 | 99 |
| **2022** | 14 | 6 | 6 | 14 | 8 | 16 | 12 | 14 | 12 | 22 | 24 | 39 | 187 |
| Lower respiratory tract infection/ Acute bronchitis (ICD J19-J20) | | | | |  |  |  |  |  |  |  |  |  |
| **2019** | 26 | 42 | 24 | 12 | 9 | 8 | 5 | 4 | 13 | 10 | 6 | 25 | 184 |
| **2020** | 22 | 17 | 16 | 3 | 3 | 1 | 9 | 6 | 9 | 11 | 5 | 5 | 107 |
| **2021** | 0 | 4 | 4 | 4 | 5 | 7 | 17 | 21 | 21 | 51 | 67 | 30 | 231 |
| **2022** | 17 | 12 | 6 | 8 | 10 | 4 | 5 | 12 | 14 | 25 | 37 | 48 | 198 |
| All Diagnosis | |  |  |  |  |  |  |  |  |  |  |  |  |
| **2019** | 62 | 95 | 59 | 31 | 28 | 23 | 26 | 19 | 43 | 39 | 23 | 65 | 513 |
| **2020** | 69 | 82 | 49 | 12 | 14 | 8 | 31 | 23 | 32 | 31 | 24 | 23 | 398 |
| **2021** | 13 | 9 | 30 | 26 | 21 | 34 | 41 | 43 | 41 | 73 | 110 | 54 | 495 |
| **2022** | 45 | 28 | 20 | 37 | 41 | 42 | 46 | 34 | 35 | 68 | 73 | 100 | 569 |

*Table E1: Numbers of patients admitted to the hospital for URTI, influenza/pneumonia or LRTI from 2019 -2022*

**Table E2:**

|  | Number of detection/ swab (n/%) | | | | | | | | | | |
| --- | --- | --- | --- | --- | --- | --- | --- | --- | --- | --- | --- |
|  | **1** | | **2** | | **3** | | **4** | | **5** |  | **Total** |
| **Virus** | **2019** | | | | | | | | | | |
| Adenovirus | 42 | 0.76 | 13 | 0.24 | 0 | 0.00 | 0 | 0.00 | 0 | 0.00 | 55 |
| Corona | 6 | 0.40 | 8 | 0.53 | 1 | 0.07 | 0 | 0.00 | 0 | 0.00 | 15 |
| Sars CoV2 | 0 | NA | 0 | NA | 0 | NA | 0 | NA | 0 | NA | 0 |
| Influenza | 191 | 0.94 | 13 | 0.06 | 0 | 0.00 | 0 | 0.00 | 0 | 0.00 | 204 |
| Metapneumovirus | 1 | 0.33 | 1 | 0.33 | 1 | 0.33 | 0 | 0.00 | 0 | 0.00 | 3 |
| Parainfluenza | 24 | 0.69 | 11 | 0.31 | 0 | 0.00 | 0 | 0.00 | 0 | 0.00 | 35 |
| Rhino | 77 | 0.76 | 23 | 0.23 | 1 | 0.01 | 0 | 0.00 | 0 | 0.00 | 101 |
| RSV | 163 | 0.89 | 21 | 0.11 | 0 | 0.00 | 0 | 0.00 | 0 | 0.00 | 184 |
| All Viruses | 504 | 0.84 | 90 | 0.15 | 3 | 0.01 | 0 | 0.00 | 0 | 0.00 | 597 |
| **Virus** | **2020** | | | | | | | | | | |
| Adenovirus | 51 | 0.68 | 21 | 0.28 | 3 | 0.04 | 0 | 0.00 | 0 | 0.00 | 75 |
| Corona (other than Sa | 7 | 0.78 | 1 | 0.11 | 1 | 0.11 | 0 | 0.00 | 0 | 0.00 | 9 |
| Sars CoV2 | 23 | 0.92 | 1 | 0.04 | 1 | 0.04 | 0 | 0.00 | 0 | 0.00 | 25 |
| Influenza | 209 | 0.94 | 12 | 0.05 | 2 | 0.01 | 0 | 0.00 | 0 | 0.00 | 223 |
| Metapneumovirus | 12 | 0.92 | 0 | 0.00 | 1 | 0.08 | 0 | 0.00 | 0 | 0.00 | 13 |
| Parainfluenza | 4 | 1.00 | 0 | 0.00 | 0 | 0.00 | 0 | 0.00 | 0 | 0.00 | 4 |
| Rhino | 249 | 0.91 | 21 | 0.08 | 3 | 0.01 | 0 | 0.00 | 0 | 0.00 | 273 |
| RSV | 102 | 0.90 | 10 | 0.09 | 1 | 0.01 | 0 | 0.00 | 0 | 0.00 | 113 |
| All Viruses | 657 | 0.89 | 66 | 0.09 | 12 | 0.02 | 0 | 0.00 | 0 | 0.00 | 735 |
| **Virus** | **2021** | | | | | | | | | | |
| Adenovirus | 42 | 0.39 | 52 | 0.49 | 13 | 0.12 | 0 | 0.00 | 0 | 0.00 | 107 |
| Corona (other than Sa | 79 | 0.61 | 39 | 0.30 | 10 | 0.08 | 1 | 0.01 | 0 | 0.00 | 129 |
| Sars CoV2 | 80 | 0.91 | 8 | 0.09 | 0 | 0.00 | 0 | 0.00 | 0 | 0.00 | 88 |
| Influenza | 4 | 0.80 | 1 | 0.20 | 0 | 0.00 | 0 | 0.00 | 0 | 0.00 | 5 |
| Metapneumovirus | 6 | 0.43 | 6 | 0.43 | 1 | 0.07 | 1 | 0.07 | 0 | 0.00 | 14 |
| Parainfluenza | 52 | 0.52 | 36 | 0.36 | 10 | 0.10 | 2 | 0.02 | 0 | 0.00 | 100 |
| Rhino | 348 | 0.70 | 128 | 0.26 | 21 | 0.04 | 2 | 0.00 | 0 | 0.00 | 499 |
| RSV | 208 | 0.69 | 80 | 0.27 | 11 | 0.04 | 2 | 0.01 | 0 | 0.00 | 301 |
| All Viruses | 819 | 0.66 | 350 | 0.28 | 66 | 0.05 | 8 | 0.01 | 0 | 0.00 | 1243 |
| **Virus** | **2022** | | | | | | | | | | |
| Adenovirus | 162 | 0.54 | 105 | 0.35 | 28 | 0.09 | 2 | 0.01 | 1 | 0.00 | 298 |
| Corona (other than Sa | 36 | 0.51 | 22 | 0.31 | 10 | 0.14 | 1 | 0.01 | 1 | 0.01 | 70 |
| Sars CoV2 | 428 | 0.84 | 64 | 0.13 | 11 | 0.02 | 3 | 0.01 | 2 | 0.00 | 508 |
| Influenza | 94 | 0.82 | 17 | 0.15 | 3 | 0.03 | 0 | 0.00 | 0 | 0.00 | 114 |
| Metapneumovirus | 43 | 0.54 | 28 | 0.35 | 7 | 0.09 | 2 | 0.03 | 0 | 0.00 | 80 |
| Parainfluenza | 114 | 0.59 | 58 | 0.30 | 17 | 0.09 | 3 | 0.02 | 2 | 0.01 | 194 |
| Rhino | 609 | 0.72 | 192 | 0.23 | 39 | 0.05 | 4 | 0.00 | 2 | 0.00 | 846 |
| RSV | 74 | 0.60 | 36 | 0.29 | 11 | 0.09 | 1 | 0.01 | 2 | 0.02 | 124 |
| All Viruses | 1560 | 0.70 | 522 | 0.23 | 126 | 0.06 | 16 | 0.01 | 10 | 0.00 | 2234 |

*Table E2: Numbers and percentage of co-infections for each virus and year*

Overall, most multiple infections were observed in younger children compared to single infections in 2022 (Figure E2).

**Figure E1:**


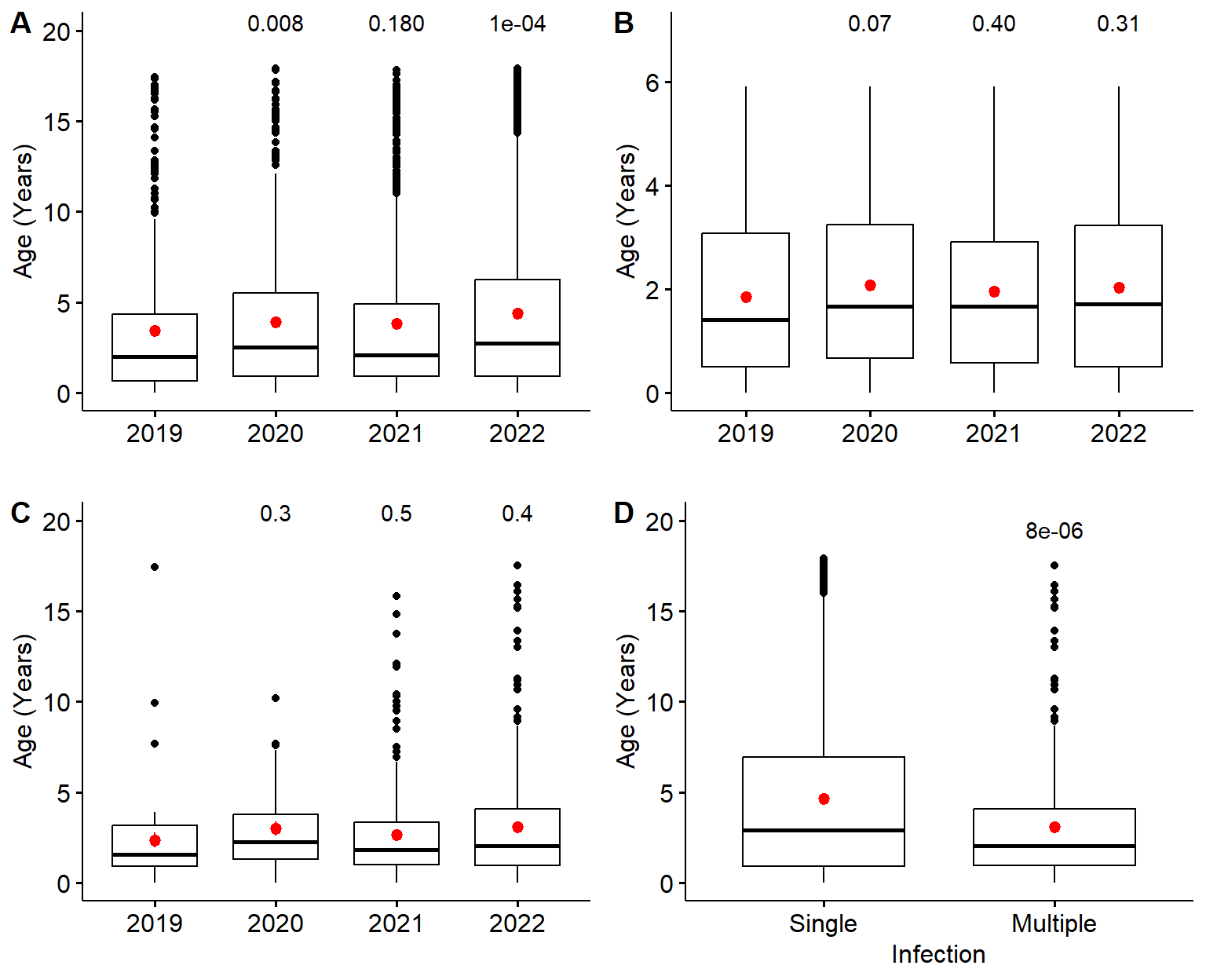


*Figure E1: Mean age per year of (A) All subjects, (B) Only single infected subjects < 6 years, (C) Only co-infected subjects, (D) Age vs co-infections 2022.*

**Figure E2:**


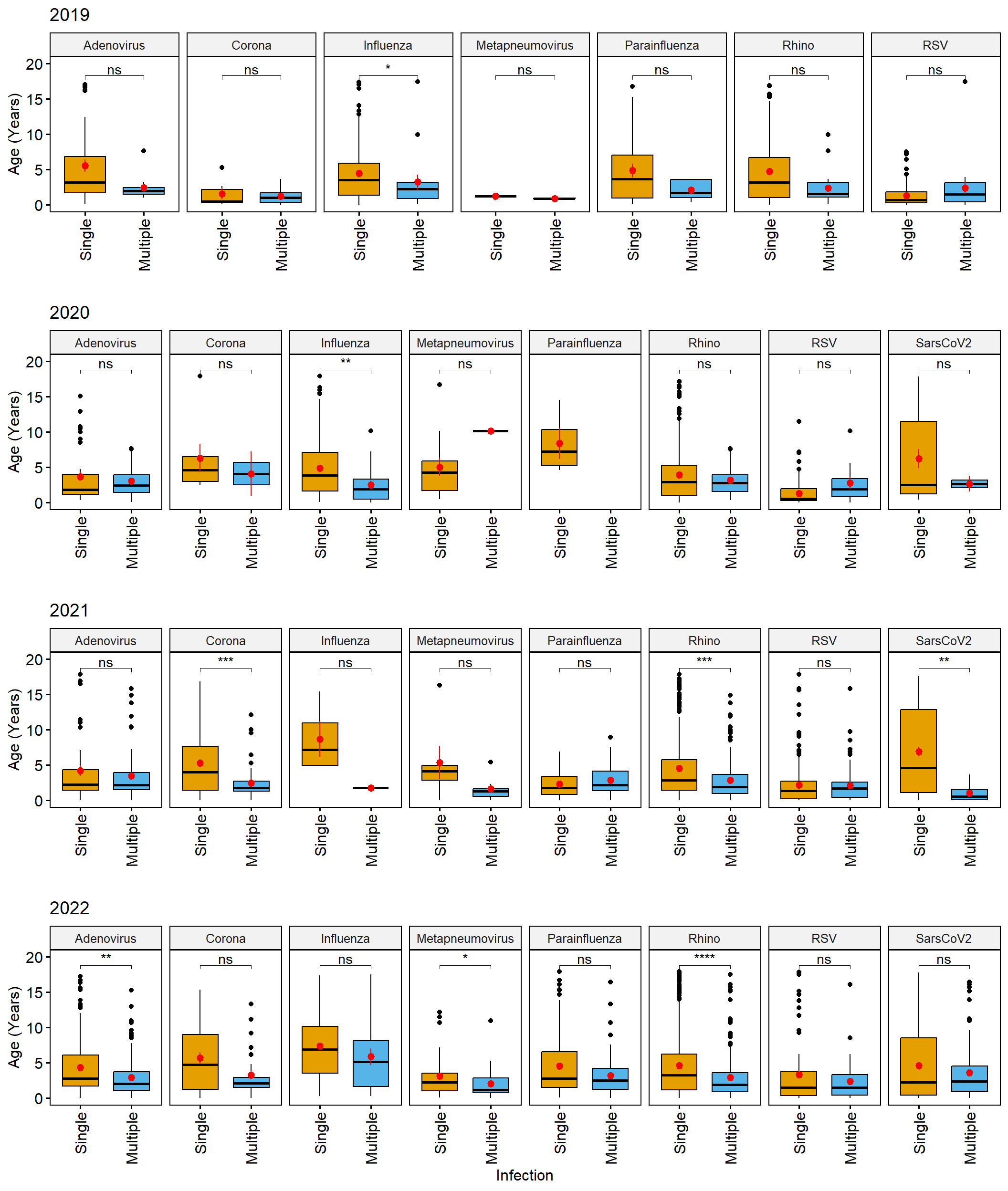


Figure E2: box plots visualizing comparison of mean age in respect to multiple and single infection for each virus and year
